# Evaluation of two prevention programs ‘Early Steps’ and ‘Faustlos’ in daycare centers with children at risk: the study protocol of a cluster randomized controlled trial

**DOI:** 10.1186/1745-6215-14-268

**Published:** 2013-08-22

**Authors:** Katrin Luise Laezer, Marianne Leuzinger-Bohleber, Bernhard Rüger, Tamara Fischmann

**Affiliations:** 1 University of Kassel, Sigmund-Freud-Institut, IDeA Center, Frankfurt am Main, Germany; 2 Institute for Statistics, Maximilian University of Munich, Munich, Germany

**Keywords:** Early childhood intervention, Prevention, Children at risk, Poverty, Socioeconomic status, Area deprivation

## Abstract

**Background:**

While early programs to prevent aggression and violence are widely used, only a few controlled trials of effectiveness of psychoanalytically based prevention programs for preschoolers have been evaluated. This study compares ‘Faustlos’ (a violence prevention program) and ‘Early Steps’ (a psychoanalytically based, whole daycare center intervention to prevent violence) in daycare centers in socioeconomically deprived neighborhoods.

**Methods/Design:**

Preschoolers in 14 daycare centers in Frankfurt, Germany, participate in a cluster randomized controlled trial (CRCT). The daycare centers were randomly chosen from a representative baseline survey of all Frankfurt’s daycare centers carried out in 2003 (*n* = 5,300) with the following stratifying factors: children’s aggressiveness, hyperactivity, anxiety and socioeconomic status. Additionally, the geographic identification of socioeconomically deprived neighborhoods regarding low-income children was taken from the Frankfurt Municipality Statistics. Children’s attachment classification and children’s aggressiveness, hyperactivity, anxiety and social competence are measured as outcome criteria before and after 2 years of intervention. The programs in the study aim to reach a high-risk population. Therefore, the combination of a random sampling of daycare centers out of a representative baseline survey in all daycare centers in Frankfurt and the application of official data on the local distribution of low-income children are unique features offered by the EVA study design. Data on preschooler’s attachment representations are collected in socioeconomically deprived neighborhoods for the first time.

**Trial registration:**

DRKS-ID: DRKS00003500

## Background

Low socioeconomic status (SES) [1-4] and neighborhood deprivation [5,6] have broad effects on children’s mental health and development. Neighborhood deprivation is associated with emotional and behavioral problems even at preschool age [7] and also disrupts children’s ability to develop school readiness [8]. Reviews of early risk factors emphasized interactive effects of aggressive behavior and delinquency as stable patterns over time with onset at early age [9].

At the same time, a growing body of research proposes that the early years of life are a particularly promising time to intervene in the lives of economically disadvantaged children, generating benefits far in excess of program costs [10-15].

In a meta-analysis, 20 early intervention programs targeting at-risk children with low-income or low-SES background have been shown to improve cognitive, emotional and behavioral, development and to reduce levels of delinquency and crime in adulthood [16]. The findings of 34 preschool prevention programs for disadvantaged children indicate positive impacts in the short, medium and long term [17,18].

Previous reviews have suggested that the following characteristics improve program effectiveness: comprehensive, multi-component programs [9,16,19-21], a duration of more than 6 months [22], and early onset in the child’s life [9,20,23,24]. Moreover, minority status (for example, ethnic background) and SES were found to be moderator variables in interventions [16,25].

The early prevention of violence, the enhancement of social learning and the support of the social integration of children were the aims of several prevention studies conducted by the Sigmund Freud Institute in cooperation with the Institute for Analytical Child and Adolescent Psychotherapy and the Municipal Education Authority in previous years, adopting the insights of these mostly US-based prevention studies. A first representative study, the Frankfurt Prevention Study (FPS), was carried out in 14 daycare centers in Frankfurt from 2003 to 2006 with children from low-SES, middle-SES and high-SES backgrounds. It was shown that aggressiveness (*P* = 0.02) and anxiety (*P* = 0.03) in boys and girls, and hyperactivity only in girls (*P* = 0.001) showed a statistically significant reduction in the prevention group in comparison to the control group [26].

The study ‘Evaluation of two prevention programs ‘Early Steps’ and ‘Faustlos’ in daycare centers with children at risk’ (EVA) compares the differential effects of two established prevention programs. Both prevention programs have been rigorously evaluated in the past [26-30]. The EVA study aims at reviewing whether the additional expenses for prevention program 1, Early Steps, is justified in the short and long term in comparison to prevention program 2, Faustlos. Taking into account one of the major findings of the large Head Start program [31], we formulated as one of our main hypotheses that, for children with a high developmental risk (for example, with a disorganized attachment pattern), the standardized, non-individualized Faustlos program will prove not to be sufficient for improving the social integration and the prevention of aggression of these children. We hypothesize that the more complex, individualized and costly Early Steps program will show positive effects on the social behavior of children at risk developmentally. Moreover, we expect the institutional setting/structure of the daycare centers to be a moderator variable. By this, we mean that the institutional setting of the daycare center (management style; participation possibilities for parents; quality of team work among teachers; fluctuation of teachers; frequency of families with multiple problems; open, half-open, or closed groups of children) influences the fit between daycare center and prevention program. For example, a daycare center with the following characteristics: a strict, top down management style, restricted/limited parent participation, high teacher fluctuation, but low frequency of families with multiple problems, might show a better fit to the Faustlos program and, therefore, might show better results in preventing aggression instead of the Early Steps program in a daycare center with the same characteristics.

## Methods

### Participants, inclusion and exclusion criteria

#### Cluster level (daycare center)

The multicenter EVA study is carried out in daycare centers with children of low-SES families who face complex, challenging problems such as unemployment, disadvantaged children, and minority status. Trying to reach children at risk with low SES, while simultaneously ensuring representativeness of the sample, we decided to use a cluster randomized controlled trial (CRCT) design in which clusters (daycare centers) are randomized.

Data from the former Frankfurt Prevention Study allowed us to draw a representative sample of all 3 to 4-year-old children in the public daycare centers of Frankfurt (5,300 children; see Table 1). Relevant stratifying variables were children’s aggressiveness, hyperactivity and anxiety as assessed by their daycare teachers as well as SES, operationalized as families’ dependency on financial support by the Frankfurt Municipality paying the monthly daycare center fee. Additionally, data from the Frankfurt Municipality Statistics on disadvantaged children in Frankfurt’s urban districts operationalized as the children’s dependency on social welfare served as a further approach to identify clusters of children at risk. Thus, we included public daycare centers in the city of Frankfurt characterized by low-SES, high-neighborhood-community risk, and frequented by children with high scores in aggressiveness, hyperactivity or anxiety.

**Table 1:** Daycare centers (DCC) and related clusters

**Factor**	**Low socioeconomic status**	**Middle/high socioeconomic status**	**Total**
Hyperactivity high Aggression high	Cluster 1: 12 DCC (5 with high anxiety scores), 6 of them selected for randomization	Cluster 2: 10 DCC (4 with high anxiety scores)	22
Hyperactivity high Aggression low	Cluster 3: 12 DCC (9 with high anxiety scores)	Cluster 4: 9 DCC (8 with high anxiety scores)	21
Hyperactivity low Aggression high	Cluster 5: 5 DCC (2 with high anxiety scores), 2 of them selected for randomization	Cluster 6: 14 DCC (5 with high anxiety scores), 1 of them selected for randomization	19
Hyperactivity low Aggression low	Cluster 7: 14 DCC (9 with high anxiety scores)	Cluster 8: 10 DCC (2 with high anxiety scores)	24
Not specified*	Cluster 9: 12 DCC, 4 of them selected for randomization	Cluster 10: 16 DCC, 1 of them selected for randomization	28
Sum of DCC	55	59	114

*Missing data: data of the ‘observation questionnaire for preschool children’ including scales of aggression, hyperactivity and anxiety could not be collected.

#### Individual level (children)

In all selected daycare centers, all of the children, male and female, between 3 years 0 months and 4 years 11 months of age at the time of recruitment and their parents were approached to participate in our program. We excluded children beyond the age range. Other exclusion criteria were not applied at the individual level.

### Interventions

The methodological requirement of random assignment gave rise to the ethical question of whether one can leave the control group without treatment and support even though the parents are struggling with serious problems concerning family life, parenting, and child development [32]. An alternative method to avoid this suggests that the control group may serve as an experimental group at a later stage of investigation. This approach was not feasible because the time frame of children staying in the daycare centers (from age 3 to 6) was too narrow. We used a second method, offered by Van IJzendoorn *et al*. [32], suggesting that intervention programs may contain several aspects that can be presented to the experimental subjects as well as to the control group without compromising the experimental design. Therefore, the EVA study compares two intervention programs: the standardized and well validated violence prevention program Faustlos, and Early Steps, a broader, more individual, prevention approach that includes Faustlos as one of its components.

#### The ‘Early Steps’ intervention

The multi-perspective and multi-component prevention program [26] Early Steps is characterized by its approach that starts with a detailed understanding of an individual child and its family. Each child is unique, as is each family. This approach is especially helpful if the specific skills and resources of the individual child are taken as the starting point. Hence, a child’s behavior is not seen primarily as ‘dysfunctional behavior’, but rather as the expression of a hidden (unconscious), reasonable, emotional development. Thus, the specific and perhaps conspicuous behavior of a child needs first to be understood and should not be simply disregarded. The aims of the supportive measures of the study are to allow the child to achieve more positive experiences of itself and its attachment figures and to develop its talents in an optimal way. Early Steps is conducted in close cooperation with the Institute for Analytical Child and Adolescence Psychotherapy. All 13 psychoanalytic therapists and supervisors in the study have at least 15 years of clinical experience in treating children and families and supervising groups (except 1 therapist with 5 years of experience). Early Steps consists of different elements:

•Biweekly case supervision of the daycare center team.

•Weekly proposal of counseling and training for educators and parents in the daycare center by experienced psychoanalytic child and adolescent psychotherapists.

•In individual cases, the proposal of therapy for children and their parents within the facilities.

•Faustlos violence prevention program in the second year of the project at the earliest.

•If required, individual mentoring of children by student teachers in their transition from kindergarten to primary school.

#### The ‘Faustlos’ intervention

This violence prevention program (literally translated: ‘without fists’) [29] is the German version of a US program called ‘Second Step’ that has been developed by the Committee for Children in Seattle and is now widely used in Germany and scientifically well researched [30]. The curriculum consists of 28 lessons and focuses on the promotion of empathy, impulse control and anger management. With the help of pictures of different emotional states, the cognition of gesture and body language of other children, as well as of oneself, is trained. Photographs that are shown to the children portray different conflict situations, which are discussed and re-enacted via roleplay. A certified educator of the Faustlos program trained the EVA daycare teachers. The implementation process of Faustlos is being documented.

### Assessments

Teachers evaluate children’s behavior (aggressiveness, hyperactivity, anxiety and social competence) and well-being using the following questionnaires: before (t0 pre), after 12 months, (t1 12 mo), after intervention (t2 post) as well as 1 year after the end of intervention (t3 follow-up): the Caregiver-Teacher Report Form 1½-5 (C-TRF) [33,34], the Strengths and Difficulties Questionnaire (SDQ) [35,36] and the Positive Development and Resilience in Daycare Center’s Daily Routine (PERIK) [37,38], an observational instrument for teachers. Moreover, the Self-Reflective Functioning Scale [39,40] will be applied for the teachers. Parent’s perspective will be evaluated using the SDQ [35,36]. Children’s assessment is conducted before (t0 pre) and after intervention (t2 post), as well as 1 year after the end of intervention (t3 follow-up). Independent and trained psychologists assess children’s intelligence using the German adaptation of Wechsler Preschool and Primary Scale of Intelligence-III Assessment (HAWIVA) [41] and as well as the children’s attachment representation. The Manchester Child Attachment Story Task (MCAST) [42], a narrative story stem task that involves playing with dolls, is a validated, structured measure that evaluates young children’s attachment representations through the use of play scenarios allowing for differentiation between four overall attachment classifications: secure attachment, insecure-ambivalent attachment, insecure-avoidant attachment and insecure-disorganized attachment representations. Measures and time points of assessment are presented in Table 2.

**Table 2:** Assessments

**Measure point**	**Child**	**Teacher**	**Parents**
t0 (pre-study)	MCAST, HAWIVA	C-TRF, SDQ, PERIK	SDQ
t1 (12 months)		C-TRF, SDQ, PERIK	
t2 (post study, 24 months)	MCAST, HAWIVA	C-TRF, SDQ, PERIK, SRS	SDQ, interview
t3 (follow-up, 36 months)	MCAST, HAWIVA	C-TRF, SDQ, PERIK	SDQ

*C-TRF* Teachers Report Form, *HAWIVA* Wechsler Preschool and Primary Scale of Intelligence-III Assessment, *MCAST* Manchester Child Attachment Story Task, *PERIK* Positive Development and Resilience in Daycare Center’s Daily Routine, *SDQ* Strengths and Difficulties Questionnaire, *SRS* Self-Reflective Functioning Scale.

### Objectives and hypothesis

The purpose of the trial is to investigate the differential efficacy of the two intervention programs in a high-risk population. The first hypothesis assumes that the program Early Steps, which individually addresses the child’s particular needs, is more effective than the standardized curriculum Faustlos in preventing aggressiveness, hyperactivity and anxiety. Secondly, we hypothesize that the more restricted approach of Faustlos might better meet the teachers’ demand for structure in some daycare centers depending on the institutional setting. Therefore, we are also interested in investigating the effectiveness of the prevention program in relation to its actual fit to the needs of the institution. Thirdly, we hypothesize that a subgroup of children in the program Early Steps will exhibit a change in their insecure attachment representation towards one that is more structured or secure.

### Design

**Figure 1:**
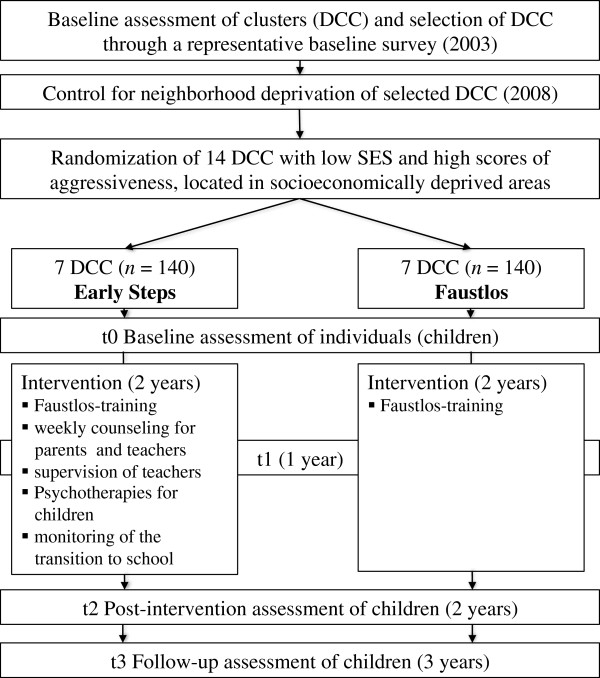
Study design.

The study is performed as a cluster-randomized controlled trial over the course of 3 years. The randomization clusters are represented by public daycare centers in the city of Frankfurt am Main, Germany that were assessed before being randomized. Figure 1 summarizes the study design.

### Outcome

The primary measure of outcome, the Manchester Child Attachment Story Task (MCAST) [42], seems to be a promising approach to evaluate results beyond the well established cognitive measures and questionnaires. Attachment representations are found to be crucial moderators for child development [43], highlighting the importance of parenting practices for children with low-SES status [4,43,44]. Therefore, the intervention response is defined as the change from children’s insecure attachment representation to a more secure one. Within the insecure attachment representations, intervention response is also defined as the change towards a more structured attachment representation (that is, statistically significant change in the disorganization score and the narrative coherence score, Likert scale, range 1 to 9).

Second outcome measures include the German adaptation of HAWIVA [41], the C-TRF [33,34], the SDQ [35,36] from the teacher’s and parent’s perspective, and the PERIK [37,38].

### Power analysis

The sample calculation and power analysis is based on α = 0.05 at a power of 0.80, using the pre/post differences of the mentioned FPS. We expect at least an effect size of *d* = 0.5. Further, if we used a RCT study design for analysis of variances, the minimum sample size would be *n* = 63 per treatment. In contrast, we applied a CRCT design with the daycare centers as given clusters. (Note: each daycare center represents one cluster.) We used a formula introduced by Eldridge *et al*. [45], providing a conservative estimate of sample size requirements for trials using cluster-level analyses weighted by cluster size.

$$n*=\left\{1+\left[\left(1+\mathrm{CV}2\right)\times m–1\right]\times \mathrm{ICC}\right\}\times n$$

The formula for the corrected sample size n* consists of the coefficient of variation *CV* for trials with unequal cluster sizes (that means with unequal sizes of daycare centers) and the intra class correlation coefficient *ICC* within the clusters and the mean cluster size *m*. Using again the findings of the Frankfurt Prevention Study, the estimated coefficient of variation is *CV* = 0.467 and the estimated intra class correlation coefficient (based on the pre/post differences including the *CV*) *ICC* = 0.0465. We expect a mean cluster size *m* = 20 or at least *m* = 17 children. The corrected sample size for the first scenario would be n* = 131.44 (if *m* = 20) divided by 20 = 6.57; in the second scenario n*=120.73 (if *m* = 17) divided by 17 n* = 7.10. For both scenarios, seven clusters, respectively seven daycare centers per treatment, should be selected.

### Representativeness of the sample and sample selection (at cluster level)

In 2003, the Sigmund Freud Institute carried out a baseline survey of all Frankfurt daycare centers (114 centers with approximately 5,300 children, aged 3 to 4). Children’s aggressiveness, hyperactivity and anxiety were measured using the teachers’ ‘observation questionnaire for preschool children’ [46]. Additionally, the SES of all daycare centers was assessed, operationalized as the family’s dependency on financial support by the Frankfurt Municipality that paid the monthly fee for the daycare center indicating low SES. These data were stratified by aggressiveness, hyperactivity, anxiety and SES and resulted in the division of 114 daycare centers into 10 clusters (see Table 1). EVA daycare centers were randomly chosen out of the clusters’ groups with low SES. (Note: here we are considering clusters on a higher level, each cluster contains several daycare centers).

### Control for neighborhood community risk (at cluster level)

**Figure 2:**
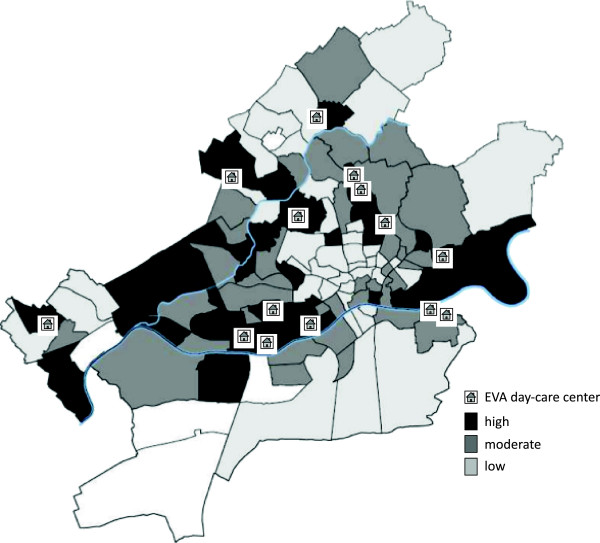
**Distribution of social welfare child beneficiaries in Frankfurt’s urban districts and position of ‘Evaluation of two prevention programs ‘Early Steps’ and ‘Faustlos’ in daycare centers with children at risk’ (EVA) study daycare centers.** Source: Jacobs [47].

To prove that our sample comprises children at risk with low SES, we additionally charted data from the Frankfurt Municipality Statistics about childhood poverty in Frankfurt’s urban districts operationalized as the children’s dependency on social welfare (in German: Sozialgeld, SGB II-Leistungsbezug, [47]). In 2009 about 20,242 children in Frankfurt under the age of 15 were on social welfare. Between the different urban districts, there are large disparities in the percentage of these children. There were districts where the percentage lay under 5% (for example, ‘West end’), which are colored in Figure 2 in a light gray, but there are other districts, where over 40% of the children were on social welfare (for example, ‘Gallus’). These quarters are colored in dark gray in Figure 2[47]. The correlation coefficient of social welfare and the unemployment rate of the parents was found to be *r* = 0.89 [47]. This high correlation hints at the cumulative geographic concentration of social problems of children in particular urban districts. The EVA daycare centers are located in these special quarters (see Figure 2).

### Randomization

As Figure 1 shows, randomization was performed at cluster level. Our statistician (TF), uninformed about the identity of the daycare centers, used a table of random numbers for randomizing the 14 daycare centers. Individual participants (children and their parents, as well as daycare teachers) were aware of their group assignment, that is, if they were participating in the Early Steps intervention or the Faustlos control group.

### Planned analyses for the primary outcome

#### Primary outcome: MCAST attachment representation

Data will be analyzed with respect to the intention of treatment. We will describe all baseline characteristics at the individual as well as at the cluster level. Moreover, we will assess the characteristics of any cluster and if the children drop out. Relevant characteristics will be added if applicable as covariates to the models. Children’s attachment representations are measured on MCAST’s overall attachment classification (secure vs insecure-ambivalent vs insecure-avoidant vs insecure-disorganized attachment representation) and on MCAST’s disorganization and narrative coherence scores. As the main analysis involves changes in MCAST disorganization and narrative coherence scores from the baseline and the endpoint in the intervention versus the control group, an analysis of covariance is applied with repeated measures and linear regression with autocorrelated data (compound symmetric covariance structure models). Changes in the overall attachment classifications are calculated by tests for frequency distributions and procedures of survival analysis (Cox, regression, log-rank tests). All procedures take the CRCT design into account. Therefore, variances of the sample mean will be corrected using the factor n*/*n* (as described above).

### Ethical issues

The Ethic Review Commission of the Federal Chamber of Child and Adolescent Psychotherapists of the State of Hessen, Germany, have approved the final study protocol and the final version of the written informed consent form. Written consent was obtained from each participating family.

### Status of the study

Ongoing recruitment.

## Discussion

The EVA study is carried out in the Center for Research on Individual Development and Adaptive Education of Children at Risk (IDeA) with financial support from the State of Hessen, Germany. One major aim of the IDeA Center is to improve the social integration and positive development of children at risk. Previous studies showed evidence of accumulation of psychosocial problems in socioeconomically deprived neighborhoods [5-8,47] and found that parenting characteristics moderate the association between SES and children’s cognitive and academic achievements [4]. Therefore, the EVA study investigates children at risk in socioeconomically deprived neighborhoods where their families face multiple challenges. Moreover, findings of attachment research influenced the EVA study design. Hence, attachment representations as crucial moderators for child development [43] highlight the importance of a ‘good enough’ early child–parent relationship experience. A secure attachment relationship between infant and caregiver is associated with optimal infant development and positive social development, including higher levels of social competence, more advanced emotional understanding and higher cognitive and language skills [48,49]. In contrast, children with an insecure-disorganized attachment representation show the highest risk later, demonstrating behavior problems that include clinical levels of externalizing and/or aggressive symptoms, hostility in the classroom as well as poor academic achievement [50-52]. This knowledge motivated us to implement an instrument in the EVA study design to classify children’s attachment representations using the MCAST. We expect to find a high- risk population regarding children’s attachment representations and to detect a high percentage of insecurely attached children. Several program modules of Early Steps (such as psychotherapy for children and their parents within the daycare center and the individual mentoring by student teachers during the transition from kindergarten to primary school) particularly address children at risk, respectively children with insecure-disorganized attachment representations. Especially for these children, we expect an improvement of their attachment representation after the individual treatment in the Early Steps group.

Since both interventions Faustlos and Early Steps have been previously evaluated and the aim of this trial is further to compare their respective effectiveness in improving behavioral and attachment related outcomes among high-risk children, we included a process evaluation in the design. The aim of this evaluation is to document the context in which the interventions were implanted and how the potentially relevant mechanisms may or may not work. We assumed that the institutional settings (for example, management style, participation possibilities for parents, quality of team work among teachers, fluctuation of teachers, frequency of families with multiple problems, open, half-open, or closed groups of children) could be determined as moderating factors for effectiveness. Therefore, regular meetings at least every 6 months were established for the directors of all participating daycare centers. During these meetings, teachers are invited to share their experiences and to discuss the ongoing process of implementing the prevention program. Regarding the Early Steps program, most questions involve considerations about the cooperation and fit between psychotherapist and teachers, psychotherapist and parents, psychotherapist and children, as well as between the supervisor and the teacher’s team. Faustlos meetings would probably concentrate on the match between the teacher administering Faustlos and the children, as well as on further adaptations of the program into the daily regime of the daycare center. In order to attain a description over time of these context and setting factors, minutes are made of the meetings. Additionally, each daycare center has at least one ‘mentor’ from the research team who will continually accompany the daycare center and their teachers during the whole intervention period and thus serve as a contact person for questions and complaints. The mentors take notes on their contacts with the personnel. The third information source of this process of implementing the program involves regular meetings between the psychotherapists and supervisors for the teams. The appraisals of these three sources are then sorted into variables, as described in the second hypothesis, in order to study their influence on the effectiveness of the programs.

One main point of interest of the IDeA Centers with high practice relevancy focuses on the professionalization of the teachers. Within the framework of this research emphasis, the EVA study assesses the impact of the team supervision for the teachers that takes place every 2 weeks in the Early Steps intervention branch, hypothesizing a process of professionalization enabling improved mentalizing and reflective processes among its teachers. We apply an instrument, the Self-Reflective Functioning Scale [39,40], which originated in attachment theory research and forms the basis for the concept of mentalizing. Fonagy *et al*. [39] define mentalizing as the ability for implicitly and explicitly interpreting the actions of oneself and others as meaningful mental states (for example, desires, needs, feelings, beliefs, and reasons). Good mentalizing in relation to other’s thoughts and feelings is characterized by the acknowledgement of opaqueness, absence of paranoia, of contemplation and reflection, perspective taking, genuine interest, openness to discovery, forgiveness, and predictability. In contrast, poor mentalizing contains an anti-reflection perspective with hostility, active evasion and non-verbal reactions, failure of adequate elaboration with a lack of integration and explanation as well as inappropriate reactions such as illogical assumptions, gross conjectures about the interviewer and taking the meanings of words literally. In our review of the process evaluation, we study the ability and the quality of mentalizing and reflection in the teachers as a prerequisite for professional work in educating children. Experiences of the Frankfurt Prevention Study [26-28] show that this professionalization takes much time to build the necessary confidence and that perhaps it may not yet have lead to the wished effects during the 2-year period of the project. In order to study the differential long-term effects of both intervention programs, we propose further follow-up studies of children in intervals between 3 and 5 years that would reach beyond the above study design.

## Abbreviations

CRCT: Cluster randomized controlled trail; C-TRF: Teachers report form; CV: Coefficient of variation; DCC: Daycare center; EVA: ‘Evaluation of two prevention programs in daycare centers with children at risk’ study; FPS: Frankfurt prevention study; HAWIVA: Wechsler preschool and primary scale of intelligence-III assessment; ICC: Intraclass correlation coefficient; m: Mean cluster size; MCAST: Manchester child attachment story task; PERIK: Positive development and resilience in daycare center’s daily routine; SDQ: Strengths and difficulties questionnaire; SES: Socioeconomic status; SRS: Self-reflective functioning scale.

## Competing interests

The authors declare that they have no competing interests.
